# The coexistence of MET over-expression and an *EGFR* T790M mutation is related to acquired resistance to EGFR tyrosine kinase inhibitors in advanced non-small cell lung cancer

**DOI:** 10.18632/oncotarget.9697

**Published:** 2016-05-30

**Authors:** Lan-Ying Gou, An-Na Li, Jin-Ji Yang, Xu-Chao Zhang, Jian Su, Hong-Hong Yan, Zhi Xie, Na-Na Lou, Si-Yang Liu, Zhong-Yi Dong, Hong-Fei Gao, Qing Zhou, Wen-Zhao Zhong, Chong-Rui Xu, Yi-Long Wu

**Affiliations:** ^1^ Southern Medical University, Guangzhou, Guangdong, People's Republic of China; ^2^ Guangdong Lung Cancer Institute, Guangdong General Hospital and Guangdong Academy of Medical Sciences, Guangzhou, China

**Keywords:** non-small cell lung cancer, EGFR-TKI, acquired resistance, MET, T790M

## Abstract

MET overexpression and the *EGFR* T790M mutation are both associated with acquired resistance (AR) to epidermal growth factor receptor tyrosine kinase inhibitors (EGFR-TKIs) in advanced non-small cell lung cancer (NSCLC). We characterized the frequency, underlying molecular mechanisms, and subsequent treatment for AR in MET overexpressing NSCLC patients with or without the T790M mutation. The study participants were 207 patients with advanced NSCLC and AR to EGFR-TKIs. The percentages of MET-, T790M- and MET/T790M-positive patients were 20.3% (42/207), 34.8% (72/207) and 6.8% (14/207), respectively. The disease control rate was 100% (5/5) for five patients with MET overexpression who received EGFR-TKIs plus a MET inhibitor. Among the MET/T790M-positive patients, seven received EGFR-TKIs plus a MET inhibitor and four received a T790M inhibitor, but no response was observed. The median post-progression survival (PPS) was 14.1, 24.5, and 10.7 months for MET-overexpressing, T790M-positive and MET/T790M-positive patients, respectively (P=0.044). c-Met, p-Met, ERBB3, and p-ERBB3 were highly expressed in MET-positive and MET/T790M-positive patients, but were poorly expressed in T790M-positive patients. EGFR, p-EGFR, AKT, p-AKT, MAPK, and p-MAPK were highly expressed in all three groups. These results suggest that MET/T790M-positive patients are at higher risk of AR to EGFR-TKIs, and have a worse PPS than patients with only MET overexpression or the T790M mutation alone. Clinical trials are needed to determine the best treatment for patients with both MET overexpression and the *EGFR* T790M mutation.

## INTRODUCTION

Epidermal growth factor receptor tyrosine kinase inhibitors (EGFR-TKIs), gefitinib, erlotinib, and afatinib,are effective therapeutic agents against non-small cell lung cancer (NSCLC) with *EGFR*-activating mutations, such as the exon 19 deletion and the L858R point mutation [1]. However, almost all tumors will develop acquired resistance (AR) to EGFR-TKIs. The main causes of AR are gatekeeper mutations in *EGFR* (the T790M second-site mutation) or bypass signaling caused by MET overexpression [2, 3].

Several strategies have been developed to overcome T790M-mediated resistance, including treatment with afatinib in combination with cetuximab, and mutant-selective EGFR-TKIs, such as CO1686 and AZD9291 [4]. Mutant-selective EGFR-TKIs have activity not only against tumors containing exon19 deletions and the L858R mutation, but also against tumors with the T790M resistance mutation [5, 6].

MET pathway activation is another mechanism of AR to EGFR-TKIs. The MET pathway can be activated in several ways, such as *MET* gene amplification, protein overexpression, activating point mutations, and induction of its ligand, hepatocyte growth factor (HGF) [7, 8]. Recently, studies reported that tumors with MET 14 exon skipping responded well to crizotinib [9–13]. However, *MET* amplification and MET 14 exon skipping are relatively uncommon phenomena. Amplification of the *MET* oncogene has been reported in approximately 5–22% of patients with AR to EGFR-TKIs [3, 14–16]. It has been suggested that a combination of the epidermal growth factor receptor (EGFR) and a MET inhibitor might be effective for overcoming resistance to EGFR-TKIs in NSCLC [3, 17]. A new MET-targeting inhibitor, INC280, has shown promising results in a phase I clinical trial reported at the 2014 American Society of Clinical Oncology meeting. This study combined gefitinib and INC280, and was used to treat *EGFR* mutant patients with AR in combination with *MET* amplification or MET overexpression [18].

Since MET overexpression and the *EGFR* T790M mutation are both important mechanisms of AR, it is important to consider MET status with or without T790M when designing clinical trials and managing clinical practice. The present study characterizes the frequency, efficacy, and molecular mechanisms of NSCLC in patients with AR and MET overexpression, with or without the *EGFR* T790M mutation.

## RESULTS

### The percentage of patients with acquired resistance to EGFR-TKIs

**Figure 1:**
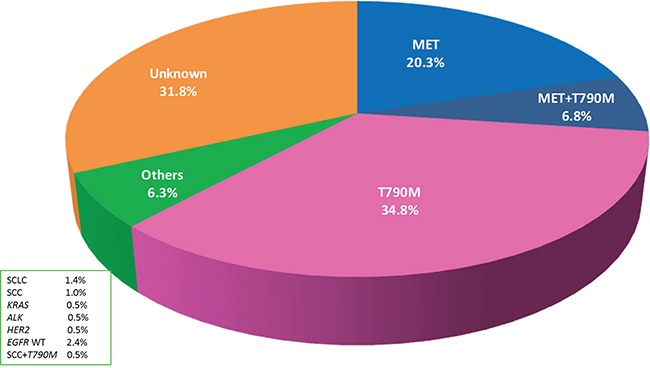
Percentages of each cause of acquired resistance (AR) to epidermal growth factor receptor tyrosine kinase inhibitors (EGFR-TKIs) in *EGFR* mutant non-small cell lung cancer (NSCLC)

From January 2013 to October 2015, 207 advanced NSCLC patients with AR to gefitinib or erlotinib were prospectively enrolled in the study (Table S1). The percentage of MET-positive patients detected by IHC was 20.3% (42/207), the percentage of *EGFR* T790M mutation patients was 34.8% (72/207), the percentage of MET/T790M positive patients was 6.8% (14/207), and the percentage of patients with additional resistance mechanisms was 6.3% (13/207). In total, 66 of the 207 (34.1%) patients had no evidence of any resistance mechanism, for which we tested in our study. The percentages of each of the resistance mechanisms are shown in Figure 1.

### Baseline clinical and molecular characteristics

The 128 patients with MET overexpression and/or T790M mutations were divided into three groups: a MET-protein overexpression group (n = 42), a T790M-positive group (n = 72), and a MET/T790M positive group (n = 14). The baseline clinicopathological and molecular characteristics of the three groups are listed in Table 1. Age, gender, smoking status, performance status, histology, *EGFR* mutation (the 19 deletion or the L858 mutation), and EGFR-TKI (gefitinib or erlotinib) were included. No differences were found in clinicopathological or molecular characteristics among the three groups. Among the 42 MET overexpression patients, 4 received EGFR-TKIs plus crizotinib, 1 received axitinib, 24 enrolled in an INC280 clinical trial (NCT01610336), 1 enrolled in a volitinib clinical trial (NCT02374645), 1 continued erlotinib, 5 received chemotherapy and the other 6 patients were lost to follow-up. Among the 72 T790M positive patients, 13 enrolled in an avitinib clinical trial (NCT02274337), 2 enrolled in an AZD9291 clinical trial (NCT02094261), 2 received AZD9291 in clinical practice, 1 received afatinib, 8 continued erlotinib or gefitinib, 33 had chemotherapy and the other 13 patients were lost to follow-up. Among the 14 MET/T790M positive patients, 7 patients received EGFR-TKIs plus a MET inhibitor and the other 7 received chemotherapy.

**Table 1:** Baseline clinical and molecular characteristics among patients that are MET protein over-expression, MET/T790M coexistence, and T790M positive

Variable category	MET positive (n=42)	MET/T790M positive (n=14)	T790M positive (n=72)	*P*
Age (median, range)	56 (32-78)	55(21-78)	54.5 (38-76)	
<65 ≥65	30(71.4%) 12(28.6%)	12(85.7%) 2(14.3%)	60(83.3%) 12(16.7%)	0.262
Gender Male Female	16(38.1%) 26 (61.9%)	5(35.7%) 9(64.3%)	30(41.7%) 42(58.3%)	0.881
Smoking status Smoker Nonsmoker	34(81.0%) 8 (19.0%)	12(85.7%) 2(14.3%)	53(73.6%) 19(26.4%)	0.486
ECOG PS ≤1 ≥2	41(97.6%) 1 (2.4%)	14(100.0%) 0(0.0%)	69(95.8%) 3(4.2%)	0.675
Histology Adenocarcinoma Squamous Adenosquamous	41(97.6%) 0 (0.0%) 1(2.4%)	13 (92.9%) 1(7.1%) 0(0.0%)	68(94.4%) 2(2.8%) 2(2.8%)	0.585
EGFR mutation DEL L858R Others	25(59.5%) 16(38.1%) 1(2.4%)	11(78.6%) 2 (14.3%) 1(7.1%)	51(70.8%) 21(29.2%) 0(0.0%)	0.141
EGFR TKIs Gefitinib Erlotinib Other EGFR-TKI	20(47.6%) 21 (50.0%) 1(2.4%)	7 (50.0%) 7 (50.0%) 0(0.0%)	43(59.7%) 29(40.3%) 0(0.0%)	0.479

ECOG PS, Eastern Cooperative Oncology Group performance status;

EGFR, epidermal growth factor receptor;

### Treatment efficacy and survival

Five patients from the MET overexpression group showed good response to EGFR-TKIs plus the MET inhibitor. One of these patients received a multikinase inhibitor, axitinib. Partial response (PR) was achieved in 80% (4/5) of the patients, one patient attained Stable Disease (SD) and all received significant clinical benefit. The longest PFS of the five patients was 7.7 months. Notably, one patient had MET overexpression in a lung lesion and an *EGFR* T790M mutation in a liver lesion. This patient achieved a mixed response: PR in the lung lesions, but a Disease Progression (PD) in the liver lesions.

Seven patients with MET/T790M coexistence received EGFR-TKIs plus a MET inhibitor, with six patients receiving first generation EGFR-TKI (gefitinib) plus a MET inhibitor, and the other patient receiving a second generation EGFR-TKI (afatinib plus a MET inhibitor). None of these treatments resulted in a response or clinical benefit. Only one patient achieved SD and PFS of 5.7 months. The other four MET/T790M positive patients received a T790M inhibitor only (avitinib) with no response. Only one patient had a longer PFS of 8.3 months (Table S2).

**Figure 2:**
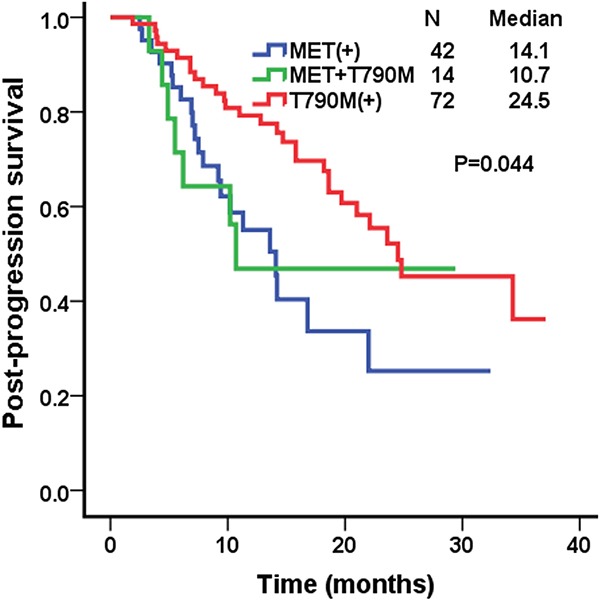
Kaplan–Meier curves for post-prognosis survival (PPS) in the MET over-expression group, the T790M positive group, and the MET/T790M positive group

Post-progression survival (PPS) was measured from the time of EGFR-TKI failure to death. The median PPS of patients with MET protein overexpression, T790M positive, and MET/T790M positive were 14.1 (95% confidence interval [CI], 10.5–17.7), 24.5 months (95% CI, 15.0–34.0), and 10.7 (95% CI, 5.6-12.4), respectively (P=0.044; Figure 2).

### Signaling pathway activity

To further investigate the molecular mechanisms of the different therapeutic effects on the three groups, several important markers of both the MET and EGFR signaling pathways were detected. Among 14 MET/T790M coexistence patients, only 9 had sufficient FFPE slides, so we can only examined the biomarkers in 9 patients from each group (Figure S2). The majority of patients were in advanced disease stage in this study, and lung biopsy was the main method used to obtain tumor specimens. c-Met, p-Met, ERBB3, and p-ERBB3 were highly expressed in the MET-protein overexpression group and the MET/T790M positive group, but poorly expressed in the T790M positive group. In contrast, EGFR, p-EGFR, AKT, p-AKT, MAPK, and p-MAPK were highly expressed in all three groups (Table 2).

**Table 2:** Protein expression of c-Met, p-Met, HGF, EGFR, p-EGFR, ERBB3, p-ERBB3, AKT, p-AKT, MAPK, and p-MAPK in tumors that over-express MET protein, T790M positive tumors, and MET over-expressing/T790M positive tumors, as determined by IHC (n=9/group)

Biomarker	MET positive (n=9)		MET/T790M positive (n=9)		T790M positive (n=9)		*P*
	n(positive)	%	n(positive)	%	n(positive)	%	
**EGFR**	9	100.0%	9	100.0%	9	100.0%	
**p-EGFR**	6	66.7%	7	77.8%	7	77.8%	0.825
**T790M**	0	0.0%	9	100.0%	9	100.0%	0.000
**MET**	9	100.0%	9	100.0%	0	0.0%	0.000
**p-MET**	7	77.8%	5	55.6%	0	0.0%	0.003
**HGF**	3	33.3%	7	77.8%	0	0.0%	0.003
**ERBB3**	5	55.6%	8	88.9%	2	22.2%	0.017
**p-ERBB3**	4	44.4%	4	44.4%	0	0.0%	0.058
**AKT**	8	88.9%	8	88.9%	7	77.8%	0.476
**p-AKT**	4	44.4%	1	11.1%	5	55.6%	0.127
**MAPK**	6	66.7%	6	88.9%	7	77.8%	0.837
**p-MAPK**	6	66.7%	7	77.8%	5	55.6%	0.607

## DISCUSSION

In the present study, we are the first to propose that MET/T790M positive patients might be more susceptible to AR towards EGFR-TKIs. Patients with both MET overexpression and the T790M mutation did not respond to a c-MET inhibitor or a T790M inhibitor alone. Based on our clinical data, albeit with a small sample size, we suggest that these patients were not suitable for clinical trials using a first generation EGFR-TKI plus a MET inhibitor (such as the INC280 clinical trial) or T790M inhibitor only. Although the frequency of MET/T790M positive patients is low (6.8%), the absolute number is large enough that more attention should be paid to this patient group. It is therefore important to consider MET status with or without the T790M mutation when managing clinical practice and designing future clinical trials.

Patients with MET overexpression that occurred after AR to EGFR-TKIs achieved a good response to EGFR-TKIs plus a MET inhibitor. Three out of four cases achieved PR and one attained a stable disease state, consistent with previous studies. Engelman et al. [3] reported that amplification of MET caused gefitinib resistance by inducing ERBB3 (HER3)-dependent activation of phosphoinositide 3-kinase [22, 23]. There are several ongoing clinical trials of MET-TKIs or MET monoclonal antibodies in combination with EGFR-TKIs, with a majority of trials selecting patients who overexpress MET. An ongoing phase I trial of a combination of INC280 and gefitinib showed good response in EGFR-TKIs resistant patients who had either *MET* amplification or MET overexpression [18]. Preclinical models also showed that EGFR-mutated cells with *MET* amplification responded to a combination of EGFR-TKIs and MET-TKIs, but were resistant to each agent given alone [24].

There is currently no clinical data on an effective therapy for patients with MET/T790M coexistence, although some preclinical investigations have recently been conducted to develop possible therapies. Bean [25] reported that a cell line, H820, contained *EGFR* mutations associated with the 19 deletion, T790M mutation, and MET amplification. These cells were resistant to erlotinib and an irreversible EGFR inhibitor, CL-387785, but sensitive to the multi kinase inhibitor, XL880. Nakagawa et al. [24], Xu et al. [26], and Nanjo et al. [27] established *in vivo* models of intrinsic resistance to reversible EGFR-TKIs with EGFRs involving the 19 deletion or the L858 and T790M mutations, as well as *MET* amplification. All of these studies found that targeting EGFR or MET alone did not produce significant tumor regression. However, a combination of a mutant-selective EGFR-TKI (a T790M inhibitor) and MET-TKI optimized both antitumor efficacy and safety. Nakagawa et al. [24] combined therapy with a next generation EGFR-TKI (WZ4002) and a MET-TKI (E7050), and Nanjo et al. [27] combined afatinib or WZ4002 with crizotinib.

To further investigate the molecular mechanisms of different responses to MET and T790M inhibitors, we selected nine cases from each group to characterize important markers of protein expression in the MET and EGFR signaling pathways. MET protein overexpression patients had markers of both EGFR and MET signaling activity. This is consistent with the clinical efficacy of first generation EGFR-TKIs combined with a MET inhibitor for the control of tumor growth in these patients. However, both EGFR and MET signaling pathways were also active in patients with MET/T790M coexistence, which might explain the clinical observations that the first generation EGFR-TKIs combined with a MET inhibitor or T790M inhibitor only were not efficacious. It might be useful to enroll MET/T790M positive patients in different clinical trials according to the markers for AR. If EGFR, p-EGFR, MET, p-MET, HGF, ERBB3, and p-ERBB3 are positive, and both the EGFR and MET signaling pathways are active, they could be enrolled in clinical trials of EGFR-TKIs (first or next generation) plus a MET inhibitor. If EGFR, p-EGFR, and T790M are positive, and only the EGFR signaling pathway is active, clinical trials involving the next generation EGFR-TKIs only (T790M inhibitor) would be recommended. In addition, if EGFR, p-EGFR, T790M, MET, p-MET, HGF, ERBB3, and p-ERBB3 are positive, and both the EGFR and MET signal pathways are active, the patients could be enrolled in clinical trials of the next generation EGFR-TKIs (T790M inhibitor) plus a MET inhibitor (Figure S3).

The majority of studies have shown that the frequency of the T790M mutation in Asian populations is 40-50% [14, 15, 28]. We also observed that T790M mutation was the most common mechanism of EGFR-TKI resistance, representing 41.6% of all cases. Among these cases, the coexistence of a T790M mutation with MET overexpression was 6.8%. However, our present results suggest that the incidence of total *MET* amplification is higher than previously reported, with 27.1% positive patients, including 6.8% patients with the T790M mutation. Past studies reported this incidence at approximately 5–22% of AR patients detected by FISH [3, 14, 15]. Notably, we found 14 patients (6.8%) with MET/T790M coexistence. The occurrence of two resistant mechanisms with a small sample size has also been reported previously. For example, among 10 EGFR-TKI-resistant tumors with MET amplification, Bean et al. reported four tumors with the T790M mutation [25]. Ji et al. [28] and Yu et al. [29] reported 7.7% (2/26) and 2.7% (2/75) patients with MET/T790M coexistence, respectively. Moreover, previous reports suggested a reciprocal relationship between EGFR T790M and MET amplification [30]. One reason that the incidence of MET positive patients was higher than in previous studies is because MET protein overexpression was detected by IHC, and samples with ≥ 50% tumor cells with high intensity staining were defined as positive in our study. This observation was based on the INC280 clinical trial, which defined MET protein overexpression as a biomarker and showed promising results [18], so more patients might benefit from MET inhibitors if overexpression of the MET protein is used as a biomarker in future studies.

The ultimate purpose of this study was to optimize treatment decisions for AR patients. We emphasized the status of two major resistance mechanisms involving MET and T790M. Both MET and T790M patients can be selectively treated, and personalized treatment strategies are available for specific types of resistance. The incidence of MET and T790M-positive patients directly reflects those who have a chance to receive the MET or T790M inhibitors. However, patients with alterations in both pathways have a worse prognosis compared with patients with only one mechanism. Consequently, it is important to design prospective clinical trials to explore treatments and overcome resistance for these MET/T790M positive patients. A major limitation of this study was that a selective EGFR-TKI (T790M) combined with a MET tyrosine kinase inhibitor was not used to treat MET/T790M positive patients. In addition, unknown mechanisms of resistance were identified in 34.1% of AR patients. It is therefore necessary that future studies include comprehensive next-generation sequencing-based mutation profiling as well as protein and gene expression analyses to identify novel mechanisms of AR.

## MATERIALS AND METHODS

### Study design and evaluation

We prospectively screened consecutive patients from January 2013 to October 2015 for *EGFR* mutations (the 19 deletion or the L858 mutation) at Guangdong Lung Cancer Institute (GLCI) Guangdong General Hospital (GGH). The study design is shown in Figure S1. We included patients with a documented clinical response to EGFR-TKI, or with a stable disease state sustained for at least 6 months. Advanced NSCLC patients with AR to EGFR-TKIs were assessed for MET overexpression and *EGFR* mutations (including T790M), as well as mutations in *KRAS*, *ALK*, and *HER2*. The 128 patients with MET overexpression and/or T790M mutation were divided into three groups: MET positive, T790M positive, and MET/T790M positive. Patients with MET protein overexpression were screened in the MET inhibitor clinical trial. Patients who were T790M positive were screened in the T790M inhibitor (such as AZD9291) clinical trial. Other patients received standard chemotherapy. c-MET, p-MET, EGFR, p-EGFR, receptor tyrosine-protein kinase erbB-3 (ERBB3), p-ERBB3, protein kinase B (AKT), p-AKT, mitogen-activated protein kinase (MAPK), and p-MAPK, which are important markers in MET and EGFR signaling pathways, were detected by immunohistochemistry (IHC). The Institutional Review Board of GLCI of GGH approved this study, and all patients provided specimens with written informed consent. Objective responses were assessed every 6–8 weeks according to the Response Evaluation Criteria in Solid Tumors (RECIST1.1). Progression-free survival (PFS) was measured from the time of treatment with the EGFR-TKI plus MET inhibitor or T790M inhibitor to disease progression. Post-progression survival (PPS) was measured from the time of EGFR-TKI failure to death.

### *EGFR* and *KRAS* mutation analyses by direct sequencing

**Figure 3:**
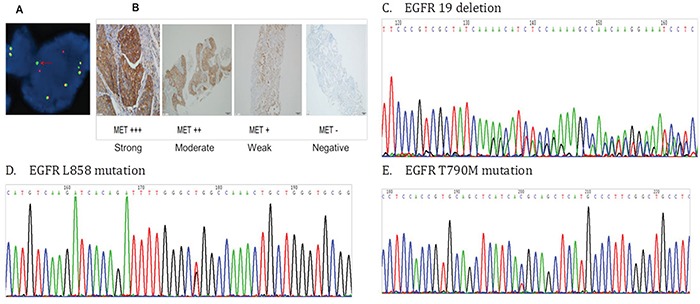
**A.** Anaplastic lymphoma kinase (ALK) fusion positive cells detected using fluorescence *in situ* hybridization (FISH) **B.** MET protein expression detected by immunohistochemistry **C.** The epidermal growth factor receptor (*EGFR*) exon 19 deletion **D.** The *EGFR* L858 mutation E. The *EGFR* T790M mutation

Genomic DNA from each sample was used for sequence analysis of *EGFR* exons 18–21 and *KRAS* exons 2–3 (Figure 3C–3E). These exons were amplified by the polymerase chain reaction (PCR) as previously described [19], and the resulting PCR products were purified and labeled for sequencing using the Big Dye 3.1 Kit (Applied Biosystems, San Francisco, CA, USA) according to the manufacturer's protocol.

### ARMS (amplification-refractory mutation system)

The ARMS method was used to evaluate *EGFR* mutation status in some cases. Genomic DNA from each sample was analyzed by Scorpion-ARMS using the *EGFR* RGQ PCR Kit or the therascreen® EGFR RGQ PCR Kit (Qiagen, Manchester, UK) according to the manufacturer's instructions. ARMS detected exon 19 deletions, L858R mutations, T790M mutations, L861Q mutations, S768I mutations, G719X mutations, and exon 20 insertions [20].

### Fluorescent in situ hybridization (FISH) assays

Interphase molecular cytogenetic studies using a commercially available *ALK* probe (Vysis LSI ALK Dual Color Break Apart Rearrangement Probe; Abbott Molecular, Abbott Park, IL, USA) were performed on 4 mm thick paraffin embedded sections. Samples were defined as FISH positive if more than 15% of scored tumor cells had split anaplastic lymphoma kinase (*ALK*) 50 and 30 probe signals or isolated 30 signals [21] (Figure 3A).

### Immunohistochemistry

IHC was performed to detect protein expression in serial sections from formalin-fixed, paraffin-embedded tumor samples, according to the protocols recommended by the manufacturer of the antibodies (Cell Signaling Technology, Danvers, MA, USA). The dilutions of c-Met, p-Met, HGF, EGFR, p-EGFR, ERBB3, p-ERBB3, AKT, p-AKT, MAPK, and p-MAPK antibodies were 1:300, 1:160, 1:40, 1:100, 1:1600, 1:250, 1:900, 1:500, 1:100, 1:100, and 1:400, respectively. MET-positive (protein overexpression) was defined as ≥ 50% tumor cells with high intensity staining (Figure 3B). HGF, EGFR, ERBB3, AKT, and MAPK positive were defined as > 50% tumor cells with moderate to high intensity staining. p-Met, p-EGFR, p-ERBB3, p-AKT, and p-MAPK positive were defined as ≥ 5% tumor cells with high intensity staining.

### Statistical analysis

The chi-square test was used to compare qualitative variables, and those with an expected frequency of < 5 were analyzed using Fisher's exact test. A value of P<0.05 was statistically significant. Kaplan–Meier curves were used to estimate PPS and OS. Data analyses were conducted using SPSS for Windows software (ver. 13.0; SPSS Inc., Chicago, IL, USA).

## CONCLUSIONS

MET/T790M positive patients might be more susceptible to AR to EGFR-TKIs, with a worse PPS than patients with only MET overexpression or the T790M mutation alone. Both MET and EGFR signaling pathways are active in these patients, and first generation EGFR-TKIs combined with MET inhibitors or T790M inhibitors alone were not efficacious for these patients. Novel treatment strategies and clinical trials are therefore needed for MET/T790M positive patients.

## SUPPLEMENTARY FIGURES AND TABLES




