# Supplementary material for: The coexistence of MET over-expression and an EGFR T790M mutation is related to acquired resistance to EGFR tyrosine kinase inhibitors in advanced non-small cell lung cancer
Source: Oncotarget. 2016 May 30;7(32):51311–9. doi: 10.18632/oncotarget.9697 (PMC5239477; doi:10.18632/oncotarget.9697)
Supplement: Supplementary file 1 [file oncotarget-07-51311-s001.pdf]

The coexistence of MET over-expression and an *EGFR* T790M mutation is related to acquired resistance to EGFR tyrosine kinase inhibitors in advanced non-small cell lung cancer

SUPPLEMENTARY FIGURES AND TABLES

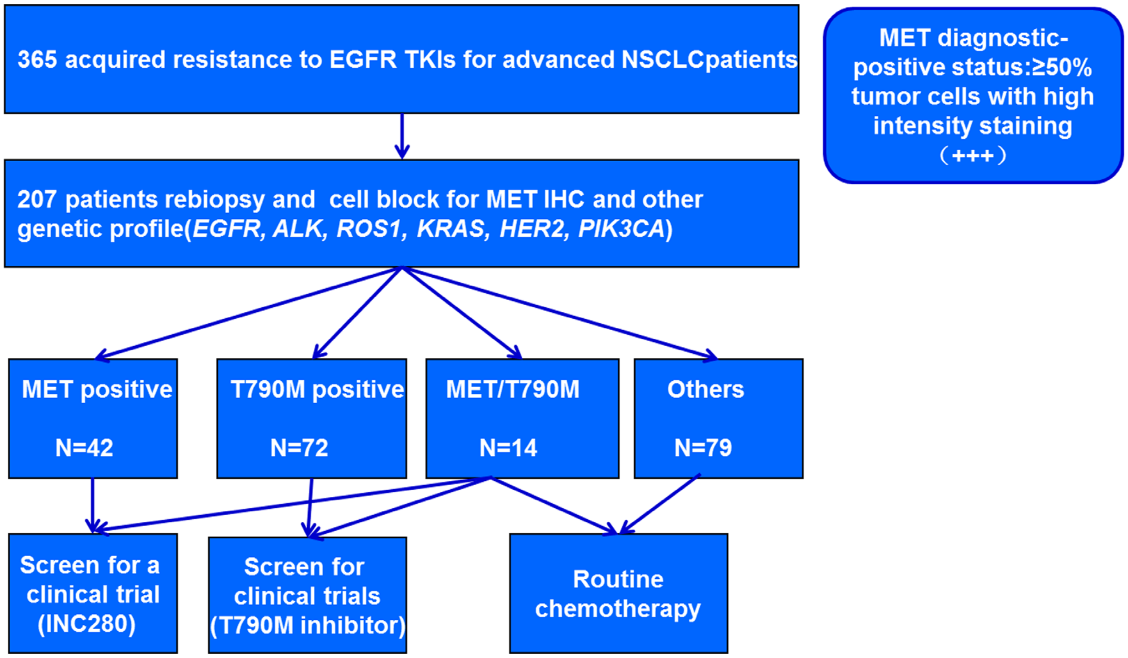

Supplementary Figure S1: Flow chart

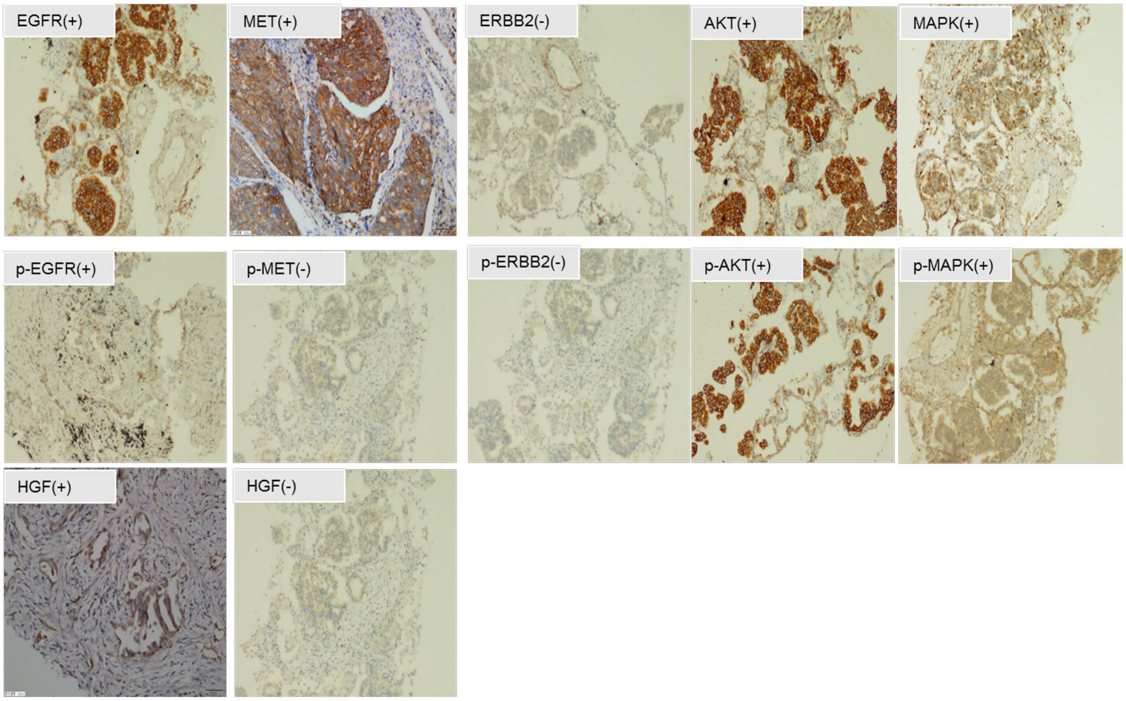

Supplementary Figure S2: Protein expression of c-Met, p-Met, EGFR, p-EGFR, HGF, ERBB3, p-ERBB3, AKT, p-AKT, MAPK, and p-MAPK in tumors

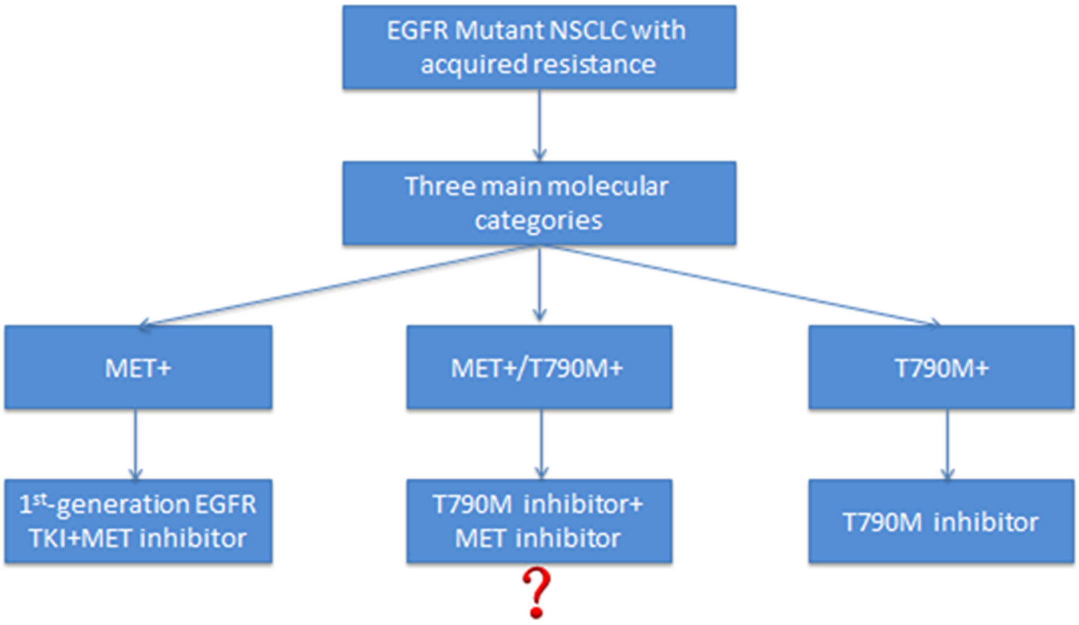

Supplementary Figure S3: Schema of overcoming AR to EGFR-TKIs in advanced NSCLC by targeting underlying molecular mechanisms

Supplementary Table S1: Baseline clinical and molecular characteristics (N=207)

Variable category	n (%)
Age (median, range)	56(21-81)
<65	159(76.8%)
≥65	48(23.2%)
Gender	
Male	91(44.0%)
Female	116 (56.0%)
Smoking status	
Smoker	161 (77.8%)
Nonsmoker	46 (22.2%)
ECOG PS	
≤1	201(97.1%)
≥2	6(2.9%)
Histology	
ADC	200 (96.6%)
SCC	3 (1.4%)
ADC with SCC	3(1.4%)
ADC with LCC	1(1.5%)
Clinical stage	
M1a IV	60 (29.0%)
M1b IV	147 (71.0%)
EGFR mutation	
DEL	130 (62.8%)
L858R	70(33.8%)
Others	7 (3.4%)
EGFR TKIs	
Gefitinib	102(49.3%)
Erlotinib	103(49.8%)
Others	1(1.0%)
MET	
Positive	56(27.1%)
Negative	161(72.9%)
T790M	
Positive	86(41.5%)
Negative	121(58.5%)

ECOG PS, Eastern Cooperative Oncology Group performance status;
EGFR, epidermal growth factor receptor.

Supplementary Table S2: Efficacy of the combination of EGFR-TKI and MET inhibitor or T790M inhibitor only for the patients with MET/T790M coexistence.

See Supplementary File 1
