# Supplementary material for: The coexistence of MET over-expression and an EGFR T790M mutation is related to acquired resistance to EGFR tyrosine kinase inhibitors in advanced non-small cell lung cancer
Source: Oncotarget. 2016 May 30;7(32):51311–9. doi: 10.18632/oncotarget.9697 (PMC5239477; doi:10.18632/oncotarget.9697)
Supplement: Supplementary file 2 [file oncotarget-07-51311-s002.docx]

| Supplementary Table S2. Efficacy of the combination of EGFR-TKI and MET inhibitor or T790M inhibitor only for the patients with MET/T790M coexistence | | | | | | | | | | | |
| --- | --- | --- | --- | --- | --- | --- | --- | --- | --- | --- | --- |
| **Patients** | **Gender** | **Age** | **Smoking** | **PS** | **Histology** | **EGFR status** | **c-MET**  **status** | **Treatment** | **Clinical benefit** | **Initial response** | **PFS**  **(month)** |
| **P 1** | F | 32 | 0 | 2 | ADC | L858R | 25%+++  30%++ | Gefitinib+ crizotinib | yes | PR | 7.5 |
| **P 2** | F | 57 | 0 | 1 | ADC | DEL | 60%++ | axitinib | yes | PR | 7.7 |
| **P 3** | F | 54 | 0 | 1 | ADC | DEL | 100%+++ | Gefitinib+ crizotinib | yes | PR | 6.1 |
| **P 4** | M | 42 | 3 | 1 | ADC | DEL | 100%++* | Gefitinib+ crizotinib | yes^#^ | SD | 4.0 |
| **P 5** | F | 50 | 0 | 0 | ADC | DEL | 60%++ | Gefitinib+ crizotinib | yes | SD | 2.0 |
| **P 6** | M | 61 | 0 | 1 | ADC | L858R T790M(liver) | 15%+++  50%++  (lung) | Gefitinib+  crizotinib | no | PD**^$^** | 1.2 |
| **P 7** | F | 76 | 0 | 1 | ADC | L858R S768I T790M | 100%+++ | Afatinib +  crizotinib | no | PD | 1.0 |
| **P 8** | M | 66 | 90 | 4 | ADC | DEL T790M | 20%+++  50%++ | Gefitinib+  crizotinib | no | PD | 1.0 |
| **P 9** | M | 63 | 40 | 1 | ADC | L858R T790M | 80%++ | Gefitinib+  INC280 | no | PD | 1.9 |
| **P 10** | M | 36 | 0 | 1 | ADC | DEL T790M | 70%+++  20%++ | Gefitinib+  INC280 | yes | SD | 5.4 |
| **P 11** | M | 55 | 30 | 1 | ADC | DEL T790M | 100%+++ | Gefitinib+  INC280 | no | PD | 0.7 |
| **P 12** | F | 69 | 0 | 1 | ADC | DEL T790M | 100%++ | Gefitinib+  INC280 | no | PD | 0.4 |
| **P 13** | M | 42 | 10 | 1 | ADC | L858R T790M | 100%+++ | Avitinib | no | PD | 1.0 |
| **P 14** | F | 47 | 0 | 1 | ADC | DEL T790M | 60%+++；30%++；10%+ | Avitinib | yes | SD | 8.3 |
| **P 15** | F | 63 | 0 | 1 | ADC | L858R T790M | 90%+++，10%++ | Avitinib | yes | SD | 4.0 |
| **P 16** | F | 44 | 0 | 1 | ADC | DEL T790M | 50%++  50%+ | Avitinib | no | SD | 2.2 |
